# Recent advances in lipid nanovesicles for targeted treatment of spinal cord injury

**DOI:** 10.3389/fbioe.2023.1261288

**Published:** 2023-08-16

**Authors:** Di Lu, Jiu-Ping Wu, Qi-Wei Yang, Hua-Yi Wang, Jun-Jie Yang, Gang-Gang Zhang, Chen Wang, Yan-Lian Yang, Ling Zhu, Xin-Zhi Sun

**Affiliations:** ^1^ Department of Orthopaedic Surgery, The First Affiliated Hospital of Zhengzhou University, Zhengzhou, China; ^2^ CAS Key Laboratory of Standardization and Measurement for Nanotechnology, National Center for Nano-science and Technology, Beijing, China; ^3^ University of Chinese Academy of Sciences, Beijing, China; ^4^ CAS Key Laboratory of Biological Effects of Nanomaterials and Nanosafety, National Center for Nanoscience and Technology, Beijing, China

**Keywords:** spinal cord injury, lipid nanovesicles, liposomes, extracellular vesicles, targeting, drug delivery

## Abstract

The effective regeneration and functional restoration of damaged spinal cord tissue have been a long-standing concern in regenerative medicine. Treatment of spinal cord injury (SCI) is challenging due to the obstruction of the blood-spinal cord barrier (BSCB), the lack of targeting of drugs, and the complex pathophysiology of injury sites. Lipid nanovesicles, including cell-derived nanovesicles and synthetic lipid nanovesicles, are highly biocompatible and can penetrate BSCB, and are therefore effective delivery systems for targeted treatment of SCI. We summarize the progress of lipid nanovesicles for the targeted treatment of SCI, discuss their advantages and challenges, and provide a perspective on the application of lipid nanovesicles for SCI treatment. Although most of the lipid nanovesicle-based therapy of SCI is still in preclinical studies, this low immunogenicity, low toxicity, and highly engineerable nanovesicles will hold great promise for future spinal cord injury treatments.

## 1 Introduction

Spinal cord injury (SCI) is a severe traumatic disease, with traffic accidents, falls, or other causes causing approximately 1 million new SCI patients each year globally (James et al., 2019). When the spinal cord is injured, information transmission from the brain to the body is disrupted or even cut off. This will result in varying degrees of loss of sensation, movement, and reflexes, as well as sphincter function below the level of the injured spinal cord segment, which can lead to paralysis. These effects can seriously reduce life quality and bring a heavy burden on society and individuals (Selvarajah et al., 2014; Shen et al., 2022). SCI has two stages: primary and secondary injury (Tator, 1995). Primary injury damages neuronal and non-neuronal cells, disrupts the blood-spinal cord barrier and the spinal cord vascular system, and causes severe damage to spinal cord tissue. Following secondary damage, a series of adverse reactions including free radical formation, calcium overload, and lipid peroxidation create a microenvironment which inhibits neural regeneration (McDonald and Sadowsky, 2002; Hayta and Elden, 2018). Many studies have shown that the key stage in SCI therapy lies in secondary injury (Park et al., 2010; Wang et al., 2019a).

Although the two stages of SCI have been explored in depth, the clinical treatment of SCI remains limited (Elizei and Kwon, 2017). Injection of methylprednisolone during the primary phase is helpful to cascade the reaction of secondary injury (Bydon et al., 2014; Bowers et al., 2016). However, high-dose methylprednisolone pulse treatment may increase the risk of adverse events such as pneumonia, infection, and gastric hemorrhage, and may contribute little to functional recovery of the spinal (Gerndt et al., 1997; Liu et al., 2019b). The conventional treatment is surgery, which involves internal fixation (Ottosen et al., 2019) and decompression (Badhiwala et al., 2021). This approach of spinal surgery creates an unobstructed recovery space for the spinal cord by removing and repairing the fractured vertebrae. Early surgical treatment provides better mitigation of secondary injury and a better prognosis (Fehlings et al., 2012). For patients with chronic spinal cord injuries, the main focus is on reducing complications and achieving self-care through rehabilitation. Physical rehabilitation can enhance the remaining muscle strength by improving the patient’s joint movement through appropriate exercise (Harvey, 2016). As a result, there is no particularly effective clinical treatment for severe spinal cord injury and recovery the patient’s damaged neurological function. In this context, researchers are beginning to shift their focus to targeted drug delivery.

Targeted drug delivery is a relatively new concept that has received increasing attention in many fields especially for nano-delivery materials targeting tumors (Khani et al., 2021) and the brain (Ravi et al., 2021). Drug targeting can be divided into passive and active targeting. Almost all drugs have a passive targeting process *in vivo*, and nanoscale particles can achieve more passive targeting with the help of EPR effect (Narum et al., 2020). Active targeting requires the assistance of a carrier to accumulate and release more of the drug in the target area while remaining stable in circulation. In general, targeted drug delivery refers to active targeting, where the drug is precisely delivered to the target cell, tissue, or organ with the binding of a receptor to a ligand. The main goal is to eliminate or reduce the accumulation of drugs in irrelevant tissues while enhancing the bioavailability of drugs at the site to be treated and prolonging the duration of drug action (Kwon et al., 2012). The lack of specific targeting makes it difficult to deliver sufficient amounts of drugs into the damaged spinal cord through traditional administration without significant side effects. In an effort to develop a drug delivery system with fewer side effects and higher bioavailability, many researchers are focusing on lipid nanovesicles as one of the best options for targeted delivery.

**FIGURE 1:**
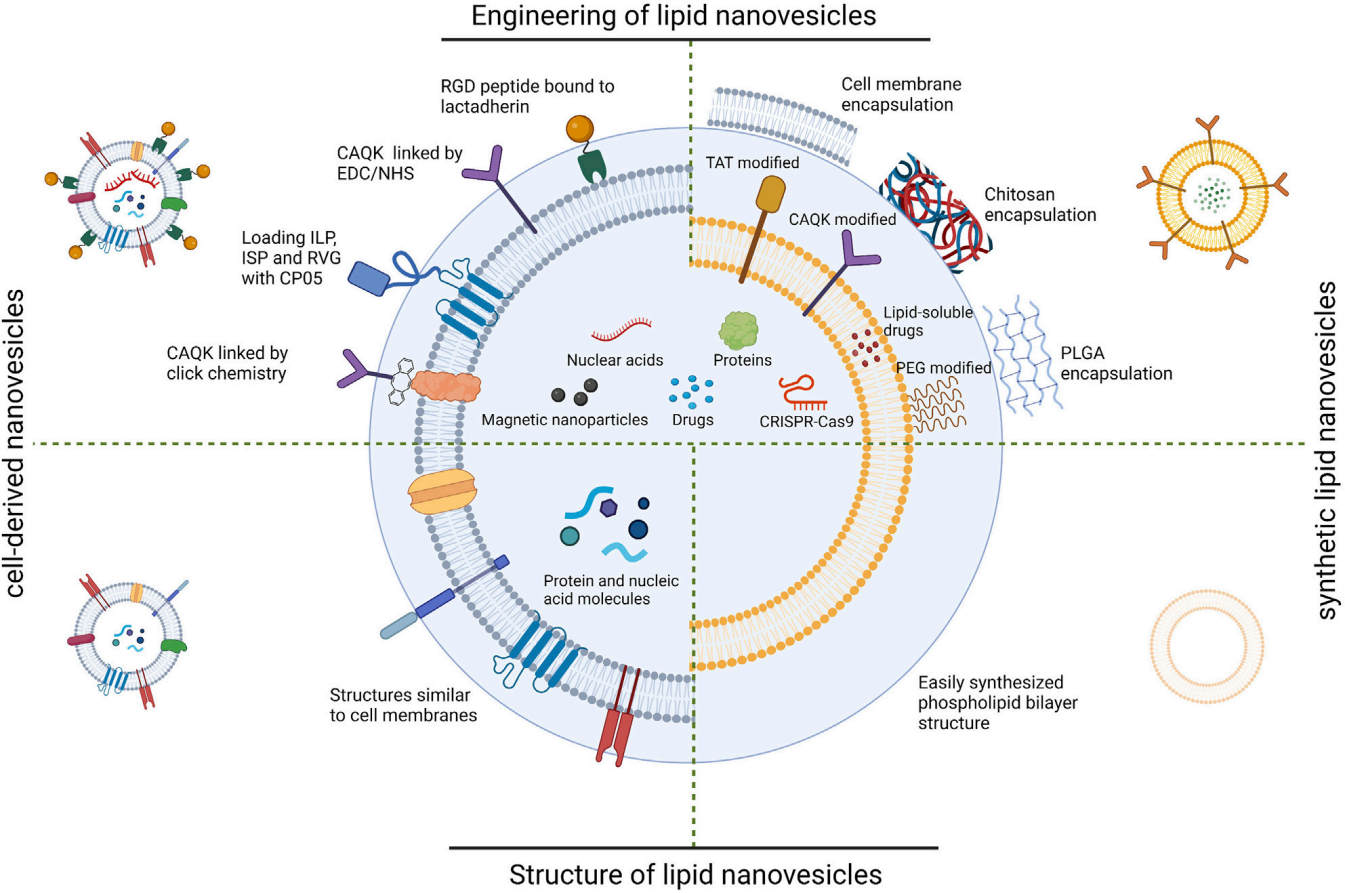
Schematic diagram of lipid nanovesicles applied to SCI targeted therapy. RGD; arginine-glycine-aspartate, CAQK; cysteine-alanine-glutamine-lysine, EDC; 1-(3-Dimethylaminopropyl)-3-ethylcarbodiimide hydrochloride, NHS; N-Hydroxysuccinimide, ILP; insulin like peptide, ISP; insulin-stimulating peptide, RVG; rabies virus glycoprotein, CP05; Cys-Arg-His-Ser-Gln-Met-Thr-Val-Thr-Ser-Arg-Leu, TAT; trans-activated transducin, PEG; polyethylene glycol, PLGA; Poly (lactid-glycolide acid). (Created with BioRender.com, accessed on 16 July 2023).

Lipid nanovesicles include synthetic lipid nanovesicles and cell-derived nanovesicles (Liu et al., 2019a). Synthetic lipid nanovesicles are phospholipid bilayer vesicle structures prepared from natural lipids or their derivatives, with diameters typically in the range of 100–200 nm (Torchilin, 2005b). Depending on the composition, they can be classified as liposomes, niosomes, transfersomes, ethosomes, etc., (Grimaldi et al., 2016a). Liposomes consisting of phospholipid bilayers are the most basic and representative of these. Due to their high biocompatibility and ability to encapsulate active molecules and controlled release of drugs, they have attracted long-standing interests in the field of drug delivery (Hallan et al., 2020; Hallan et al., 2021). Moreover, the modification of liposomes can be easily achieved by different lipid components or surface modifications (Wang et al., 2021b). Over the last few decades, efforts have been made to develop liposome-based drug delivery systems. Many drug candidates are encapsulated in liposomes and have been investigated for reducing toxicity and prolonging the therapeutic effects (Filipczak et al., 2020). For cell-derived lipid nanovesicles, their composition is similar to the cell membrane of the cells that produce them. Depending on the source, they can be categorized as extracellular vesicles, cell membrane vesicles, exosome-mimicking nanovesicles, etc., Cell-derived lipid nanovesicles can transport a variety of active molecules into target cells and participate in various physiological and pathological processes *in vivo*, completing the regulation of cellular biological functions and intercellular information transfer processes (Jeppesen et al., 2019). These nanovesicles are highly biocompatible, easy to prepare, specific biodistribution, and selective targeting capabilities, as well as improved stability (Hung and Leonard, 2015). Representatives of them, extracellular vesicles (EVs), has good biocompatibility and a stable ability to circulate *in vivo*. Therefore, EVs may be an ideal drug carrier for SCI therapy (Fu et al., 2020). On this basis, an increasing number of investigators have begun to use lipid nanovesicles for the targeted treatment of SCI. (Figure 1).

In this review we first present the challenges of targeted drug delivery to the spinal cord and then describe the preparation methods, properties, and delivery modalities of different lipid nanovesicles and their application for the target treatment of SCI. Considering that there are some commonalities in treating diseases related to the central nervous system, we have also interspersed some studies on brain-targeted therapies that may be of value. Finally, we outline the current status and challenges of using lipid nanovesicles for targeted therapy and discuss possible directions for subsequent research.

## 2 Challenges and solutions to spinal drug delivery for SCI

The spinal cord is one of the most challenging organs for drug delivery. To achieve successful targeting, the first hurdles that need to be overcome are crossing the blood-spinal cord barrier (BSCB). Secondly, controlling the delivery of drugs to injury sites at the desired frequency, duration, and concentration is another major difficulty.

### 2.1 Crossing the BSCB

The BSCB is the most important interface for molecular exchange between blood and spinal cord parenchyma. Similar to the blood-brain barrier (BBB), the barrier function of the spinal cord capillaries is based on a specialized system of vascular endothelial cells and their accessory structures, including basement membranes, pericytes, and astrocyte terminal peduncles. Coordination between these building blocks facilitates BSCB regulatory and protective functions and makes it the primary barrier of the spinal cord (Bartanusz et al., 2011). Despite morphological and functional differences between the BSCB and BBB and reduced barrier function during spinal cord injury, the BSCB remains a major barrier to spinal cord drug delivery (Lee et al., 2017). Firstly, the tight junctions reduce the penetration of ions and other hydrophilic substances through the intercellular space, forming a “physical barrier”. Secondly, extracellular pumps, such as P-glycoprotein in endothelial cells, transfer metabolic wastes and other foreign substances from the spinal parenchyma to the bloodstream, forming a “transport barrier”. Extracellular and intracellular enzymes in the spinal parenchyma metabolize many toxic foreign substances, forming an “enzyme barrier” (Jin et al., 2021). All these barriers result in less efficient penetration of drugs into the spinal cord. Administration by intrathecal injection allows the drug to enter the spinal cord through the cerebrospinal fluid circulation. However, this invasive procedure may cause infection (Austin et al., 2012). Pharmacological approaches focusing on drug modification, such as carrier-mediated and receptor-mediated transport, are still unsatisfactory for the permeation efficiency of BSCB. (Juillerat-Jeanneret, 2008). Despite Difficulties, emerging nano-delivery systems hold great potential in penetrating the BSCB. The small size of nanoparticles allows them to be translocated to endothelial cells by endocytosis and then enter the parenchymal tissues of the spinal cord via cytosol. (Kong et al., 2012). Interactions between ligands on the surface of nanoparticles can occur with endothelial cell receptors, which can trigger plasma membrane invagination and contraction to form vesicles, which are translocated through receptor-mediated transcytosis (Israel et al., 2020). Some nanoparticles can also open tight junctions between endothelial cells leading to local permeability of the BSCB(Freese et al., 2014). As part of the nanoparticles, lipid nanovesicles, with relatively higher biocompatibility and circulating stability, as well as the ability to penetrate BSCB and target SCI by themselves or with modifications, have more potential for SCI therapy (Morad et al., 2019).

### 2.2 Targeting delivery and combinational treatment

As the connection between the central nervous system to the peripheral nervous system, the spinal cord is complex in composition, consisting of various cell types, including neurons and glial cells (including microglia, oligodendrocytes, and astrocytes), ventricular canal cells, and endogenous stem cells (McDonald and Sadowsky, 2002). One way to improve the efficiency of drug delivery is the need to incubate drugs with the ability to target specific cells. Moreover, it is found that different parts of the brain and spinal cord showed different permeability to the cytokines such as interferon-α(IFN-α), interferon-γ(IFN-γ), and tumor necrosis factor-α(TNF-α) (Pan et al., 1997). The dose administered needs to be adjusted to the specific site and extent of the injury to achieve precise treatment. On the other hand, the pathophysiological mechanisms of SCI involve inflammation, microenvironmental changes, apoptosis, and scar formation. The current therapeutic agents for SCI often target one of the pathological processes. Delivery systems may need to carry multiple drugs to achieve a combined therapeutic effect. Faced with this problem, several researchers are beginning to work on responsive or temporal and spatial drug delivery designs for nanomedicine delivery systems. The expectation is to achieve responsive drug release or integrated therapeutic treatment for inflammatory sites of spinal cord injury (Xu et al., 2023; Zuo et al., 2023). The high degree of engineerability makes lipid nanovesicles good modifiable platforms.

## 3 Advantages of lipid nanovesicles for the targeted treatment of SCI

**FIGURE 2:**
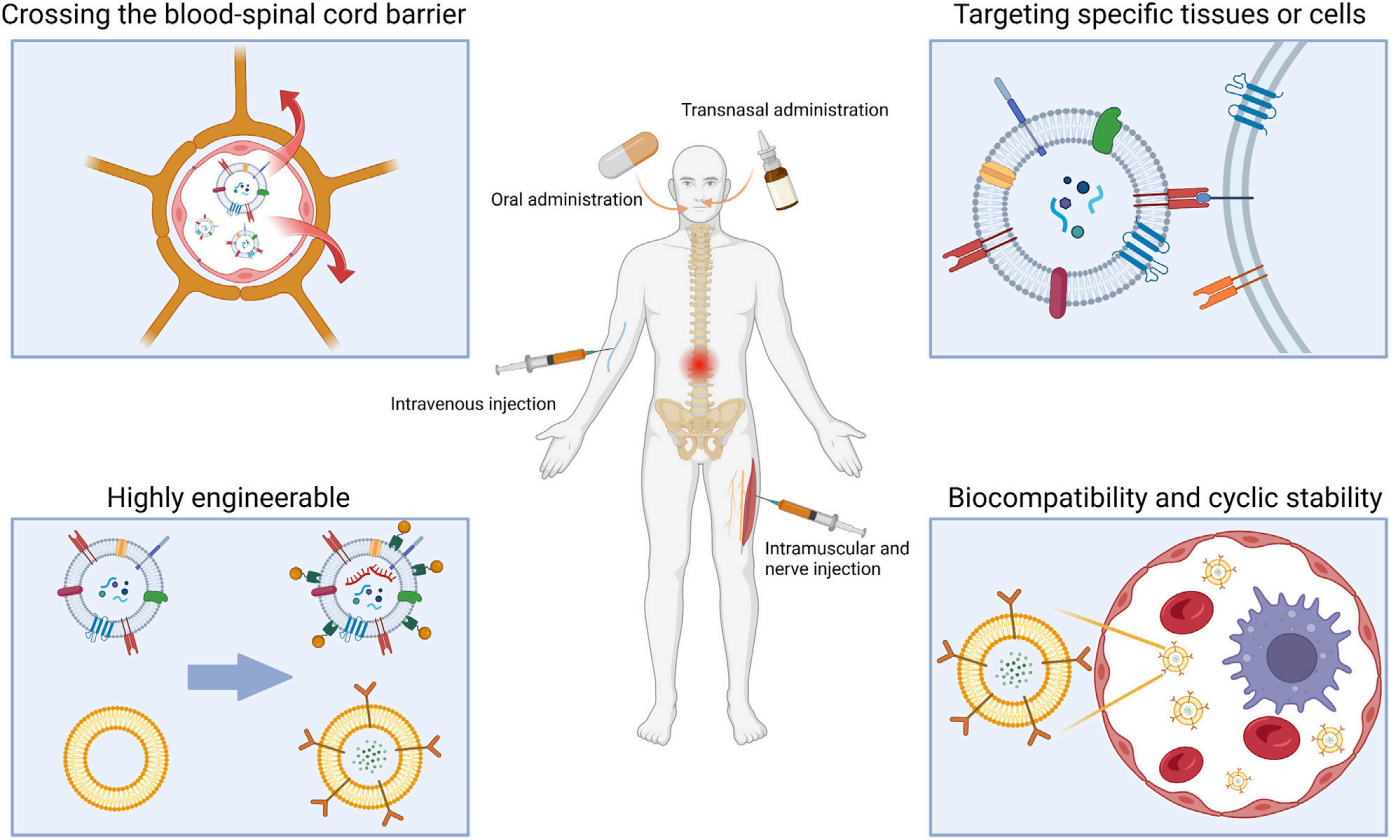
Advantages and various drug delivery modes of lipid nanovesicles for SCI traetment. (Created with BioRender.com, accessed on 16 July 2023).

Despite the challenges, there has been some progress in SCI therapy. Some drugs are in clinical trials, but efficacy and mechanisms need to be further elucidated (Kunte et al., 2015). Recent cellular therapies for SCI have reaped some benefits, including *in situ* transplantation or intravenous infusion of cells or stem cells. However, it still faces safety and ethical issues (Zipser et al., 2022). The use of hydrogel scaffolds to guide nerve regeneration has also made some progress, there is hope of reconnecting the severed spinal cord with the help of scaffolds. Nevertheless, the biocompatibility, fine structure, and subsequent degradation of implants could be improved (Walsh et al., 2023). On the other hand, nano drug delivery system brings new approaches to therapeutics. Compared to conventional drugs, nano-delivery systems have lower liver and kidney toxicity (Stanwick et al., 2012). The large specific surface area allows most drugs encapsulated in nanocarriers to be located at or close to the particle surface for faster release (Singh et al., 2009). However, the complete process of nanomaterials *in vivo* remains unknown (Bobo et al., 2016), especially for metal and inorganic nanoparticles, which sometimes exhibit enhanced biological toxicity (Love et al., 2012). In addition, nanoparticles larger than 200 nm may activate the complement system leading to an inflammatory immune response (Mahmoud et al., 2020). The size of the nano delivery system also affects BSCB penetration efficiency, with nanoparticles of approximately 50 nm in diameter being more easily internalized than other sizes (Lu et al., 2009). Furthermore, the charge should also be a consideration in targeted therapy, with positively charged nanoparticles disturbing the integrity of the blood-brain barrier (Lockman et al., 2004). To avoid disruption of the BSCB, nanoparticles with a negative zeta potential are more suitable for drug delivery to the spinal cord. Most lipid nanovesicles are negatively charged and have a particle size of around 50–200 nm. Compared to other nanoparticles, this phospholipid-based structure has lower immunogenicity and toxicity and is more accessible to the spinal cord. The hydrophilic cavity of lipid nanovesicles can also be used to load metallic or inorganic nanoparticles, increasing their biocompatibility and delivery efficiency (Ke and Afonin, 2021). With specific receptors or modifications on the surface, lipid nanovesicles can also achieve better *in vivo* cycling stability and longer cycling time (Kamerkar et al., 2017). On top of that, lipid nanovesicles are already available for traditional intravenous and oral delivery, as well as for emerging transnasal delivery and intramuscular or neurological injection. Researchers have modified lipid nanoparticles to enable more effective SCI-targeted therapy through different delivery methods. (Figure 2).

## 4 Synthetic lipid nanovesicles for targeted treatment of spinal cord injury

Almost all of the synthetic lipid nanovesicles currently used for SCI therapy are obtained by further processing and modification on the basis of liposomes. Liposomes are artificial vesicles made from natural phospholipids and cholesterol. (Akbarzadeh et al., 2013). Over the decades, researchers have attempted many modifications to conventional liposomes, including resizing, changing the ratio of raw materials, and making surface modifications to improve their drug delivery capabilities (Grimaldi et al., 2016b). Liposome formulations for cancer drug delivery have achieved significant clinical and commercial success, such as Doxil^®^, a liposome formulation encapsulated in DOX (Barenholz, 2012). There have also been lots of advances in the field of targeted therapy for SCI. In the 1990 s, researchers found that cells had greater uptake of positively charged liposomes (CL), spatially stable liposomes (polyethylene glycol liposomes), while negatively charged liposomes (AL) were barely taken up by cells (Miller et al., 1998). The targeting specificity of cationic liposomes on neovascular endothelial cells was subsequently demonstrated (Krasnici et al., 2003). In rats with experimental autoimmune encephalomyelitis, the BBB is liability bound by CL. Moreover, CL exhibits sensitive, rapid, and efficient accumulation in the affected intra-neural vessels, with accumulation directly correlating with the degree of inflammation. Anionic liposomes do not have this function. Oxidation resistance 1 plasmid (pOXR1)-loaded cationic liposomes injected into the T9-T10 SCI site in rats alleviated oxidative stress and promoted functional recovery by activating the nuclear factor erythropoietin-2-related factor 2/heme oxygenase 1(Nrf2/HO-1) pathway (Zhang et al., 2021). These studies suggest that synthetic lipid nanovesicles may be a candidate vehicle for targeted drug transport to sites of inflammatory lesions in the spinal cord (Cavaletti et al., 2009). Next, we will present studies related to synthetic lipid nanovesicles in SCI-targeted therapies based on different modification methods and innovative delivery approaches (Table 1).

**TABLE 1:** Synthetic lipid nanovesicles for SCI targeted therapy.

Name	Ingredients	Size	Animal	Mechanism of targeting	Targeting sites	References
TAT-PEG-MPL	PEG-OQCMC, Chol, Superparamagnetic nanoparticles, TAT	avg. 83.2 nm	SD rats	External magnetic fields and TAT efficiently crossing BSCB	Enriched in spinal cord lesions	Wang et al. (2010)
PLGA/CsA NPs	PEG-OQLCS, TAT-OQLCS, Chol, PLGA, CsA	251.4 ± 2.2 nm	Wistar rats	TAT efficiently crossing BSCB	Enriched in spinal cord lesions	Gao et al. (2017)
RM-LIP	PC, Chol, mPEG 2000-DSPE, Minocycline, Macrophage membrane	110.08 ± 1.97 nm	Mice	Inflammatory tropism	Enriched in spinal cord lesions	Tang et al. (2021)
MH-DS@M-Lips	Soy phosphatidylcholine, Chol, MH, DS, Macrophage membrane	120 nm–150 nm	C57BL/6J mice	Inflammatory tropism	Enriched in spinal cord lesions	An et al. (2022)
CAQK-LIP-BDNF/DTX	Phospholipids, Chol, Docetaxel, BDNF, CAQK	176.29 ± 5.34 nm	SD rats	CAQK peptides targeting CSPGs at injury sites	Enriched in spinal cord lesions	Wang et al. (2018)
PEG-liposome	DOTAP, DOPC, DOPS, Chol, DSPE-PEG2000	80–90 nm	Mice	Transnasal administration allows PEG-modified and electroneutral liposomes reach the brain and spinal cord	Enriched in brain and spinal cord	Singh et al. (2018)
CTB-R8-DSPC	DSPC, Chol, DSPE-PEG2000, CTB, R8	125.00 ± 6.16 nm	Lewis rats	Reverse transport from peripheral nerves or muscles to the spinal cord	Enriched in spinal cord	Fukui et al. (2022)
COL-SA-Lip/CUR	HSPC, COL, NHS-PEG2000-DSPE, Se-apamin, CUR	avg. 122.5 nm	BALB/c mice and SD rats	Neuropeptide apamin targeting astrocytes	Intestinal EGC, astrocytes in spinal cord lesions	Wang et al. (2021c)

Abbreviations: PEG-OQCMC, PEGylated amphiphilic octadecyl quaternised carboxymethyl chitosan; Chol, Cholesterol; SD, Sprague-Dawley; CsA, Cyclosporine A; NPs, nanoparticles; OQLCS, octadecyl-quaternized lysine modified chitosan; PC, phosphatidylcholine; mPEG, 2000-DSPE, N-(Carbonyl-methoxypolyethylene glycol 2000)-1,2-distearoyl-sn-glycerol-3-phosphoethanolamine; MH, minocycline hydrochloride; DS, dextran sulfate; BDNF, brain-derived neurotrophic factor; DOTAP, (2,3-Dioleoyloxy-propyl)-trimethylammonium-chloride; DOPC, 1,2-dioleoyl-sn-glycero-3-phosphocholine; DOPS, 1, 2-dioleoyl-sn-glycero-3-phospho-L-serine; DSPC, 1,2-Dioctadecanoyl-sn-glycero-3-phophocholine; CTB, cholera toxin B; R8, Octa-arginine; HSPC, 1,2-Diacyl-sn-Glycero-3-Phosphocholine; COL, chitosan oligosaccharide lactate; Se-apamin, diseleno bond-stabilized apamin; CUR, curcumin.

### 4.1 Trans-activated transducin (TAT)-modified liposomes

**FIGURE 3:**
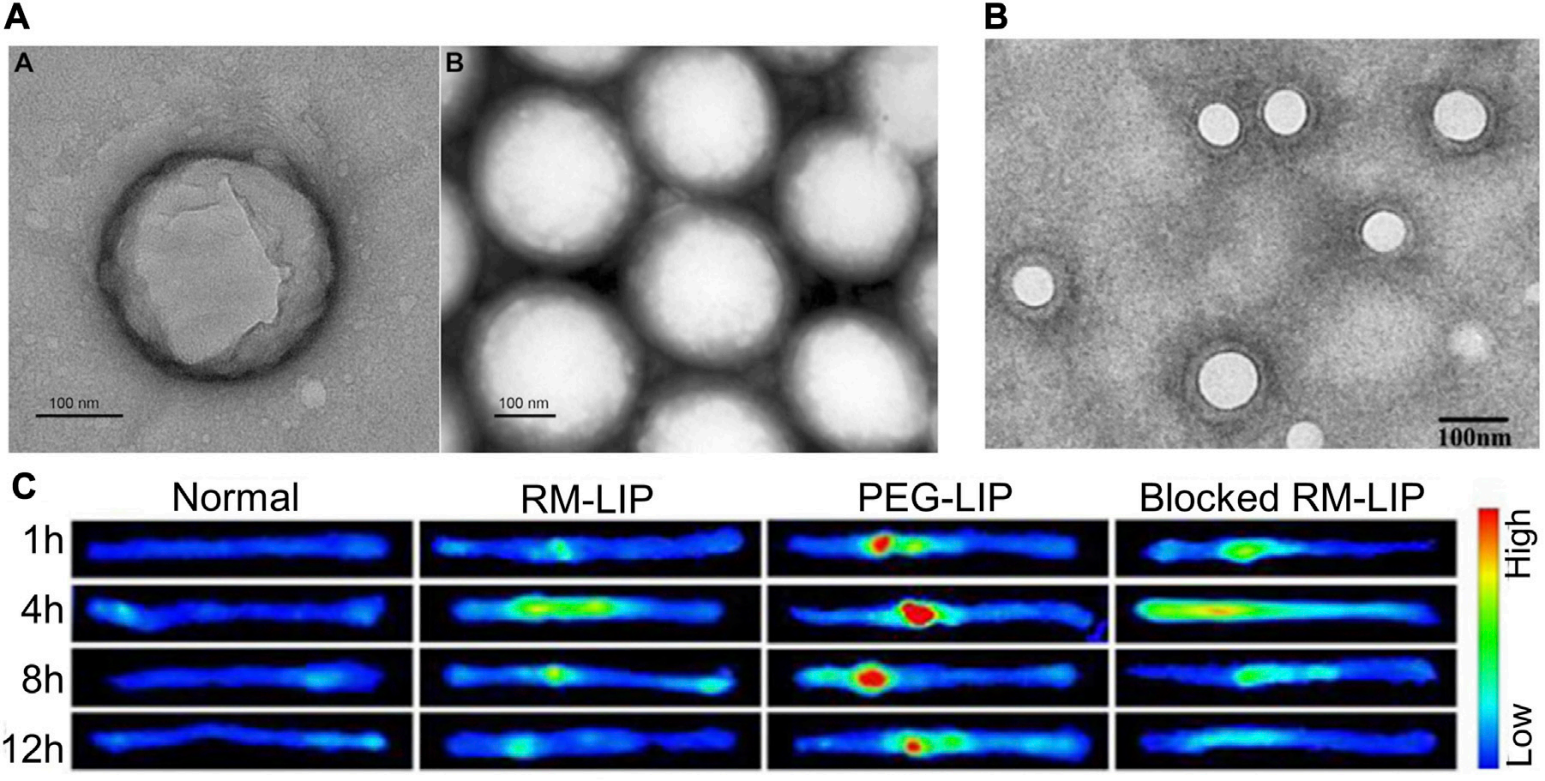
Partial results of research on synthetic lipid nanovesicles for the targeted treatment of SCI. **(A)** TEM image of PLGA/CsA NP. Reprinted with permission from Ref. (Gao et al., 2017). Copyright ^©^ *2017 Taylor & Francis.* **(B, C)** TEM image of RM-LIP and its powerful targeting ability to SCI sites. Reprinted with permission from Ref (Tang et al., 2021). Copyright *© 2021, Elsevier*.

TAT is a polypeptide consisting of 86 amino acids that can efficiently pass through cell membranes to exert biological effects (Said Hassane et al., 2010). Liposomes can cross cell membranes when they are attached to TAT under its mediation (Torchilin, 2005a). This provides a viable pathway for cell membrane transduction. TAT can assist in targeted therapy for SCI. TAT-PEG conjugated magnetic polymeric liposomes (MPL) were developed combining the benefits of polymeric vesicles and liposomes, with TAT giving these liposomes the ability to cross the BSCB (Wang et al., 2010). In addition, PEGylated amphiphilic octadecyl quaternised carboxymethyl chitosan (PEG-OQCMC) prevents particle aggregation and encapsulates large amounts of drugs through spatial and electrostatic repulsion. Hydrophobic superparamagnetic nanoparticles can be guided by magnetic fields to confer MPL targeting and act as contrast agents for MRI. PLGA/liposomes modified with PEG-TAT and containing cyclosporin A can cross the BSCB and target aggregation around the contused spinal cord of rats (Gao et al., 2017) (Figure 3A). The hydrophobic poly (lactic acid)-glycolic acid copolymer (PLGA) core provides efficient drug encapsulation for sustained release of cyclosporin A (25 h). This controlled drug delivery and release offer the possibility for clinical application.

### 4.2 Macrophage membrane camouflaged liposomes

Given the simple lipid structure of liposomes does not provide sufficient advantages in terms of stable circulation and targeted transport *in vivo*, the researchers have developed biomimetic nano-delivery system. The transfer of bioactive components to the surface of liposomes allows for a higher level of active targeting and biocompatibility while retaining the physical properties of conventional liposomes (Xuan et al., 2016; Ji et al., 2019; Li et al., 2019). Macrophage membrane-decorated liposomes (RM-LIP) can be prepared by liposome extruders (Tang et al., 2021) (Figures 3B, C). These nanovesicles assisted in enhancing the targeting of minocycline to SCI sites in mice after tail vein injection. This is because RM-LIP can specifically bind to inflammatory endothelial cells. Macrophage membrane coating reduces nanoparticle uptake by macrophages and prolongs *in vivo* circulation. This study also found that the levels of Mac-1 and integrin-α4 in macrophages as well as the targeting of RM-LIP to SCI were independent of macrophage polarization status. The same approach was used to construct MH-DS@M-Lips, a liposome wrapped by primary macrophage membranes and loaded with minocycline hydrochloride (MH) and dextran sulfate (DS). MH-DS@M-Lips also have a powerful inflammatory targeting ability and a more comprehensive SCI therapeutic effect (An et al., 2022). In brain injury treatment, liposomes loaded with baicalein (BA-LP) were also surface-modified with macrophage membranes for the treatment of middle cerebral artery occlusion (MCAO) rats (Long et al., 2022). The method of imparting better targeting and biocompatibility of liposomes by wrapping the cell membrane deserves more extensive research.

### 4.3 Other surface modified liposomes

More peptides are used to modify liposomes to enhance targeting or therapeutic effects against SCI. CAQK-LIP-BDNF/DTX are liposomes loaded with doxorubicin (DTX) and brain-derived neurotrophic factor (BDNF) with cysteine-alanine-lysine glutamine (CAQK) peptides modified on the surface. With the ability of CAQK to target damaged nerves, This lipid nanovesicle can deliver two drugs targeted to the rat SCI site via blood circulation (Wang et al., 2018). Another peptide with antioxidant capacity, glutathione, has been shown to facilitate drug transport through the BBB by the assistance of glutathione transport proteins on the BBB. The use of GSH-PEG-modified liposomes (2B3-201) allowed for targeted delivery of MP to acute experimental auto lesions in rats with immune encephalomyelitis (EAE) (Gaillard et al., 2012). On this basis, the therapeutic efficacy of 2B3-201 against myelin oligodendrocyte-induced experimental autoimmune encephalomyelitis (MOG-EAE) in mice has also been demonstrated (Lee et al., 2014). Similar efficacy was obtained with only one-tenth of the treatment with high-dose free MP and without complications. Considering the persistence of inflammation during SCI, the targeting of 2B3-201 or GSH-PEG liposomes carrying other drugs to SCI also deserves to be explored.

### 4.4 New attempts in liposome administration methods

In the decades-long study of liposomes, scientists have also tried a range of non-intravenous drug delivery techniques. One of the hot topics is central nervous system targeting through intranasal delivery. Liposomes loaded with Allium cepa fraction can cross BBB and protect nerves at the site of ischemic stroke (Singh et al., 2018). Recent studies have found that PEG-modified neutral liposomes have higher efficiency of intranasal delivery to the central nervous system (CNS) than CL and AL (Kurano et al., 2022). This electrically neutral liposome allows drug delivery to the brain and spinal cord via the trigeminal pathway. Since PEG modification decreases cellular uptake efficiency, future researchers could make a breakthrough in imparting positive charges or modifying peptides on the surface of liposomes (dos Santos Rodrigues et al., 2020; Feng et al., 2021).

Retrograde transport from peripheral tissues to the spinal cord using adenovirus via axons is another new mode of drug delivery (Wang et al., 2021a). However, viruses have low delivery efficiency and can only be loaded with nucleic acid-based drugs. Therefore, some researchers have attempted to develop liposomes that can be administered from peripheral nerves or muscles and can be delivered to the spinal cord by optimizing the composition or modifying the surface. Recent studies have found that liposomes composed of DSPC, choline, and PEG lipids can be reverse-transported from peripheral nerves or muscles to the spinal cord (Fukui et al., 2022). In addition, the uptake of this liposome by neurons could be enhanced by modifying R8 and CTB, and CTB also significantly improved the efficiency of retrograde axonal transport. CCTB, which is part of the cholera toxin, can efficiently enter the axon through GM1 receptors (Zuilhof, 2016), while R8 consists of eight arginines with good cell permeability (Khalil et al., 2008). With the aid of these two non-cytotoxic proteins, the retrograde transport of liposomes in axons for targeted delivery has great potential.

Oral administration is the most convenient route of drug delivery, but research on transoral targeted drug delivery systems is not yet satisfactory due to the gastrointestinal environment and barrier function (Sadeghi et al., 2020). Enteric glial cells (EGCs) are an important component of the enteric-central nervous system axis, resembling astrocytes in morphology, function, and biomarker expression, and may even be directly involved in the pathology of CNS(Klingelhoefer and Reichmann, 2015; Ochoa-Cortes et al., 2016). Based on this commonality, researchers chose conventional liposomes as carriers for loading CUR and covered them with a protective layer composed of non-covalent cross-linked chitosan oligosaccharide lactate (COL) and modified with diseleno bond-stabilized apamin (Se-apamin). They named this liposome as COL-SA-Lip/CUR (Wang et al., 2021c). Apamin is a ligand for small conductance calcium-activated potassium (SK) channels expressed on both astrocytes and EGCs (Wu et al., 2014), and its stability is enhanced by diselenium bond substitution. COL provides a temporary protective layer for liposomes and promotes permeation of the intestinal mucosa after dissociation (Tang et al., 2018). C-SA-Lip/CUR exhibited excellent oral bioavailability in the duodenum, jejunum, and ileum following oral administration to rats with SCI in T10 segments. It exhibited up to 12 h of accumulation in damaged spinal cord sites, co-localizing with astrocytes. Efficient absorption and glial cell targeting after oral administration resulted in reduced inflammation and accelerated recovery of the damaged spinal cord. At the same time, the intestinal dysfunction brought about by SCI was also alleviated. This more convenient and comprehensive form of targeted therapy deserves further development.

## 5 Cell-derived lipid nanovesicles for targeted treatment of spinal cord injury

Compared to liposomes, cell-derived lipid nanovesicles, especially extracellular vesicles, are much younger members in the drug delivery field. EVs are cell-derived, phospholipid-based bilayer particles, rich in proteins, lipids, nucleic acids, and other biologically active substances, widely present in various body fluids such as blood and urine (Keller et al., 2011; Jeppesen et al., 2019). EVs can be broadly classified into two categories, exosomes (exos) and ectosomes. Exosomes are EVs with endosomal origins ranging in size from 40 to 160 nm in diameter. Ectosomes are vesicles that interrupt the surface of the plasma membrane by outward budding and include microvesicles, microparticles, and large vesicles ranging in size from approximately 50 nm to 1 mm in diameter (Kalluri and LeBleu, 2020). The majority of EVs described in this review are exosomes.

EVs have low immunogenicity and permeability to biological barriers, they can also deliver information to target cells through direct action, endocytosis, or interaction with cell membranes, and participate in physiological processes such as cell cycle, proliferation, and apoptosis (Vlassov et al., 2012). The homing and migratory properties inherited from secretory cells give EVs the ability to target specific tissues or organs (Mulcahy et al., 2014). On this basis, researchers have attempted to use chemicals or proteins to modify EVs or load them with drugs to create engineered EVs with increased targeting or more functionality (Tian et al., 2018). Therapeutic agents or molecules can be packed into EVs either by intracellular co-expression or by electroporation-based drug loading. For instance, miRNA-124-3p can be loaded into neural stem cell-derived exosomes (NSC-exos) using electroporation, and NSC-exos successfully delivered miR-124-3p into glioma cells and inhibited tumor growth (Qian et al., 2022). Targeting peptides or antibody fragments can be decorated on the surface of EVs to confer cell and tissue specificity (Liang et al., 2021). Researchers have used metabolic glycoengineering to obtain dextran sulfate-modified exosomes (DS-exos) from adipose stem cells. After intravenous administration to mice with collagen-induced arthritis (CIA), DS-exos effectively targeted inflamed joints, modulating the macrophages population in the synovium and promoting the regression of arthritis. Similarly, many EVs have been used to target SCI (McHugh, 2021). We summarize the progress of cell-derived lipid nanovesicles, represented by EVs, in SCI targeted therapy according to different cell sources (Table 2).

**TABLE 2:** Cell-derived nanovesicles for SCI targeted therapy.

Source	Size	Animal	Preprocessing	Mechanism of targeting	Targeting sites	References
BMSCs	59.09 ± 0.58 nm	SD rats	-	Inflammatory tropism	Macrophages, astrocytes and neurons at SCI sites	Lankford et al. (2018)
BMSCs	avg. 150 nm	SD rats	Loading miR-494	Inflammatory tropism	Neurons at SCI sites	Huang et al. (2021)
BMSCs	-	SD rats	Overexpression of miR-133b	Inflammatory tropism	Not verified	Li et al. (2018a)
hucMSCs	30–150 nm	SD rats	Overexpression of miR-146a-5p	Inflammatory tropism	Astrocytes at SCI sites	Lai et al. (2022)
BMSCs	50–100 nm	SD rats	Overexpression of miR-26a	Inflammatory tropism	Neurons and neurons at SCI sites	Chen et al. (2021)
BMSCs	-	SD rats	Overexpression of miR-145-5p	Inflammatory tropism	Not verified	Jiang and Zhang (2021)
ADSCs	avg. 123.5 nm	SD rats	Hypoxia treatment	Inflammatory tropism	Enriched in spinal cord lesions	Liang et al. (2022)
BMSCs	121.6–125.3 nm	C57BL/6 mice	Hypoxia treatment	Inflammatory tropism	Microglia and macrophages at SCI sites	Liu et al. (2020)
hucMSCs	50–150 nm	C57BL/6J mice	Surface modification with CAQK peptides and contain CRISPR/Cas9 components	CAQK peptides targeting CSPGs at injury sites	Enriched in the injured sites	Wang et al. (2022)
iNSCs	avg. 120 nm	C57BL/6 mice	Surface modification with CAQK peptides and contain siRNA	CAQK peptides targeting CSPGs at injury sites	Enriched in the injured sites	Rong et al. (2023)
hBMSCs	50–150 nm	SD rats	Loading with PTEN siRNA	Neuroinflammation-mediated chemotaxis	Enriched in spinal cord lesions	Guo et al. (2019)
hMSCs	avg. 150 nm	mouse	Loading with IONPs	External magnetic fields	Enriched in spinal cord lesions	Kim et al. (2018)
hucMSCs	233.5 ± 70.3 nm	C57BL/6 mice	Macrophage membranes fusion with hucMSCs to obtain MF-NVs after extrusion	Inflammatory tropism and increased adhesion to ischemic endothelial cells	Enriched in spinal cord lesions	Lee et al. (2020)
Mouse primary macrophage	125 ± 12 nm	C57BL/6J mice	Loading Ber	Inflammatory tropism	Macrophages, microglia and neurons at the site of injury	Gao et al. (2021)
RAW264.7	130.4 ± 28.8 nm	C57BL/6 mice	Macrophage membranes loaded with NGF by extrusion	Inflammatory tropism	Enriched in spinal cord lesions	Xia et al. (2021)
RAW264.7	134 ± 11 nm	C57BL/6 J mice	macrophage membranes loaded SA and NAL by extrusion	Inflammatory tropism	Enriched in spinal cord lesions	Liu et al. (2022)
Mouse primary microglia	113.4 ± 12.1 nm	SD rats	Loading Res	Inflammatory tropism	Enriched in spinal cord lesions	Fan et al. (2020b)
Plasma	About 80–140 nm	C57BL/6 mice	Surface modified by three peptides: RVG, ILP and ISP	Enhanced homing of AP-EXO to spinal cord neurons by RVG binding to p75NTR	Most colocalized with neurons at the injury	Ran et al. (2022)

Abbreviations: BMSCs, bone marrow stromal cells; miR, microRNA; hucMSCs, human umbilical cord mesenchymal stem cells; ADSCs, adipose stem cells; CSPG, chondroitin sulfate proteoglycan; iNSCs, induced neural stem cells; siRNA, small interfering RNA; hBMSCs, human bone marrow stem cells; PTEN, phosphatase and tensin homolog; IONPs, iron oxide nanoparticles; MF-NVs, macrophage-derived nanovesicles; Ber, berberine; NGF, nerve growth factor; SA, sodium alginate; NAL, naloxone; Res, resveratrol; p75NTR, neurotrophin receptor p75.

### 5.1 Stem cell-derived EVs

Stem cell-related research has long been a priority for scientists in the field of regenerative medicine. (Zakrzewski et al., 2019). The most current research for SCI therapy has focused on EVs secreted by mesenchymal stem cells (MSCs) (Qu and Zhang, 2017; Liu et al., 2021). MSCs are innately low in immunogenicity and can also tend to localize to sites of inflammation when attracted by chemokines and adhesion factors (Kidd et al., 2009). Initially, researchers found that in immunosuppressed rats, tail vein injected MSCs could be transplanted to the sites of SCI (Morita et al., 2016). However, in non-immunosuppressed rats, no intravenous MSCs were detected at SCI sites (Quertainmont et al., 2012; Matsushita et al., 2015). This phenomenon limits the application of MSCs. Further studies revealed that the therapeutic effect of MSCs was mainly achieved with the assistance of their secreted exosomes (Zhang et al., 2015). Compared to MSCs, exosomes from MSCs(MSC-exos) are easier to obtain and store as well as being virtually ethically unrestricted (Gimona et al., 2017). Being significantly smaller in size than MSCs, exosomes are not trapped by lung and liver tissue and can penetrate the BSCB(Wang et al., 2019b). A series of studies have shown that MSC-exos can repair injured spinal cord by anti-inflammatory, anti-apoptotic, promoting angiogenesis (Huang et al., 2017), inducing axonal regeneration (Li et al., 2018b), and enhancing the BSCB (Lu et al., 2019). Recent studies have also found that MSCs injected intravenously into rats do not reach SCI sites, but can release exosomes and be taken up by M2-type macrophages at injury lesions (Nakazaki et al., 2021). Similarly, after intravenous administration of bone marrow MSCs exosomes (BMSC-exos) to rats with SCI at the T9 level, BMSC-exos was abundantly enriched in the injured region and taken up by M2-type macrophages, while almost absent in the normal spinal cord. They also found that BMSC-exos could simultaneously reach the spleen and assist in the recovery process of SCI by modulating the overall immune system function (Lankford et al., 2018). In addition, the bioinformatic analysis showed that MSC-exos contains a large number of miRNAs targeting the Toll-like receptor (TLR)4/NF-κB signaling pathway (Fan et al., 2020a).

**FIGURE 4:**
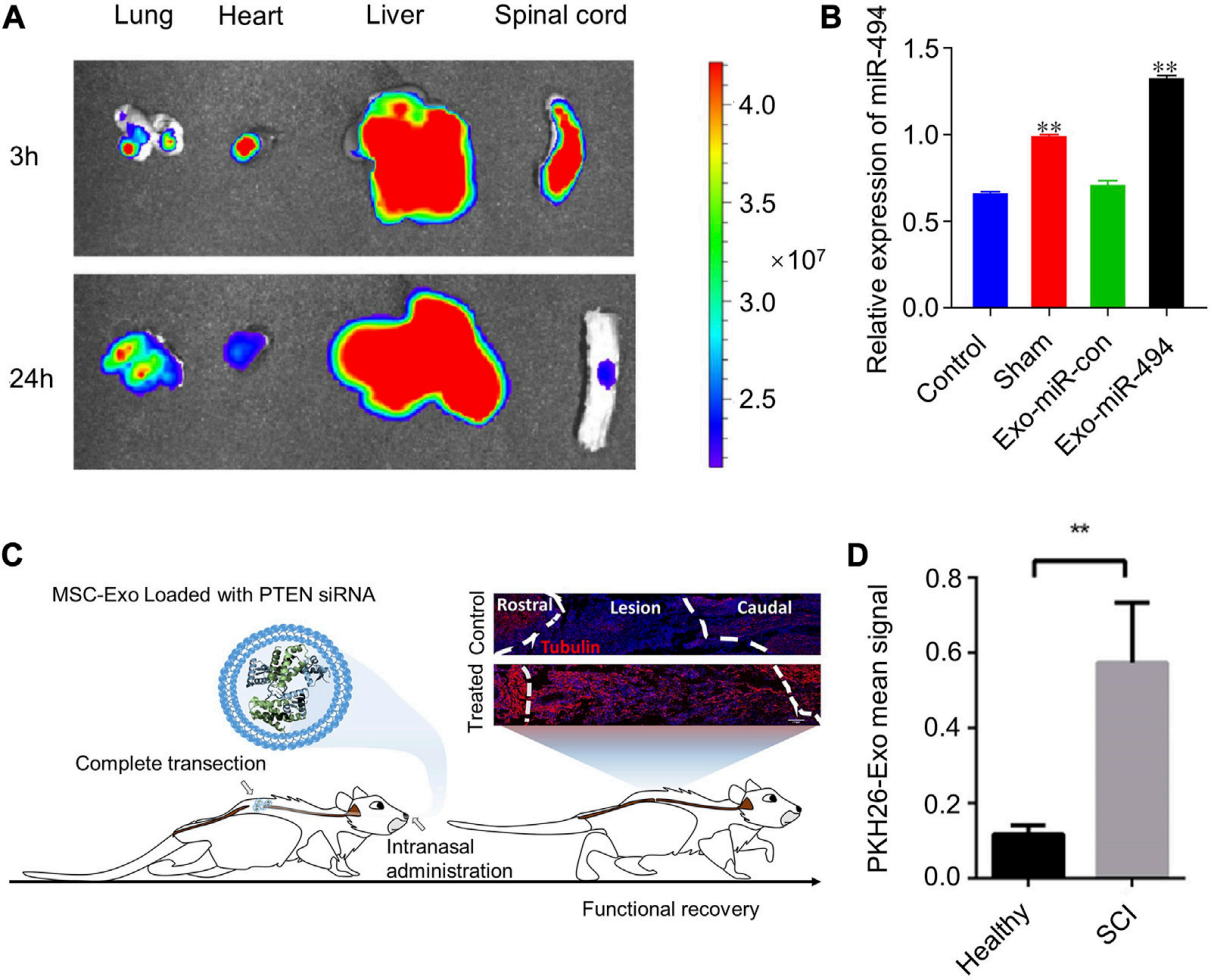
Examples of targeted therapy for SCI with the help of extracellular vesicles. **(A,B)** MSC-Exo was enriched in rat SCI sites and elevated miR-494 expression levels. Reprinted with permission from Ref. (Huang et al., 2021). Copyright *© 2021 Wei Huang et al.* **(C, D)** MSC-Exo loaded with phosphatase and tensin homologue small interfering RNA (PTEN-siRNA) for transnasal targeting to regions of SCI. Reprinted with permission from Ref. (Guo et al., 2019). Copyright *© 2019, American Chemical Society.*

Based on the inflammatory targeting of MSC-exos, we can use them as candidate drug carriers to SCI sites. By chemical transfection, miR-494 was loaded into rat bone mesenchymal stem cell-derived exosomes (Exo-miR-494), and Exo-miR-494 was taken up by rat liver and nerve cells at the injury sites after tail vein injection. (Huang et al., 2021). (Figures 4A, B) Together with miR-494, exosomes improve the local immune environment and promote the recovery of motor function. Similarly, multiple miRNA, such as miR-133b and miR-26a etc., are loaded with MSC-exos for SCI therapy (Li et al., 2018a; Chen et al., 2021; Jiang and Zhang, 2021; Lai et al., 2022). More interestingly, exosomes obtained from adipose stem cells after hypoxic treatment had higher miR-499-5p content. Such exosomes can also have better targeting effect on SCI (Liang et al., 2022). Hypoxia-treated BMSCs secreted EVs with higher miR-216a-5p content. Such EVs could shift microglia at SCI sites from M1 pro-inflammatory phenotype to M2 anti-inflammatory phenotype by inhibiting TLR4/NF-κB and activating the PI3K/AKT signaling pathway (Liu et al., 2020).

**FIGURE 5:**
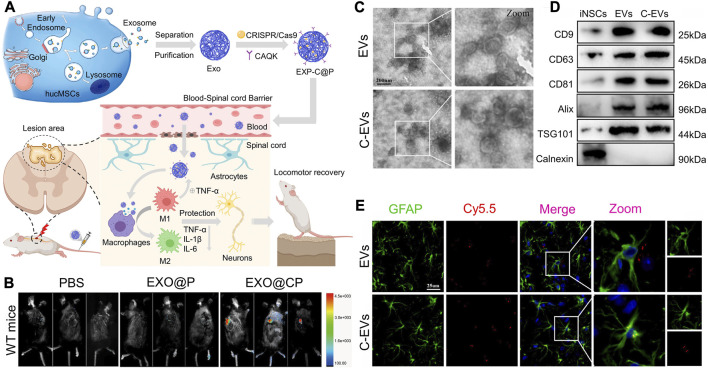
Surface modification of cell-derived lipid nanovesicles to enhance their ability to target SCI. **(A,B)** Schematic representation of engineering hucMSCs to form EXP-C@P and the distribution of targeting damaged spinal cord. Reprinted with permission from Ref. (Wang et al., 2022; Copyright ^©^ 2022, Elsevier. **(C,D)** Morphology and marker proteins of EVs and c-EVs derived from iNSC. **(E)** Co-localization analysis of Cy5.5-labeled EV and Cy5.5-labeled C-EV with astrocytes. Reprinted with permission from Ref. (Rong et al., 2023; Copyright ^©^ 2023, Elsevier.

Surface modification of MSC-exos can improve the targeting ability to SCI. To make emerging gene editing therapies more applicable to SCI therapy, researchers used CAQK peptides to modify human umbilical cord MSCs secreted exosomes (hucMSC-exos) and loaded CRISPR/Cas9 plasmids into the exosomes (EXO-C@P). This peptide selectively binds to the proteoglycan complex in injured neural tissue (Mann et al., 2016; Wang et al., 2018), which can help EXO-C@P specifically target SCI sites (Wang et al., 2022). (Figures 5A, B) The released CRISPR/Cas9 plasmid genetically edited the activated immune cells such as macrophages, T cells, and neutrophils, thereby inhibiting the TNF-α-induced inflammatory response and promoting recovery from SCI. CAQK is also immobilized on the membrane of neural stem cell-derived EVs by a copper-free click chemistry method, helping EVs to target monocyte chemoattractant protein-1(CCL2)-siRNA to the site of SCI (Rong et al., 2023). (Figures 5C–E) A recombinant fusion protein, containing an arginine-glycine-aspartate (RGD)-4C peptide (ACDCRGDCFC), was fused to the phosphatidylserine (PS) binding domain of lactadherin (C1C2). This recombinant protein can easily self-bind to the membrane of EV secreted by human neural progenitor cells, forming RGD-EV (Tian et al., 2021). After intravenous administration, RGD-EV targets lesioned areas of the ischemic brain.

Another form of non-invasive central nervous system drug delivery—intranasal delivery—has been the focal point of many researchers in central nervous system CNS disorders. Drug delivery studies in brain disorders have demonstrated that s can cross the BBB by intranasal administration (Zhuang et al., 2011; Perets et al., 2018) and are better retained at the injury lesion compared to intravenous administration (Haney et al., 2015; Betzer et al., 2017). On this basis, phosphatase and tensin homologous small interfering RNA (PTNA-siRNA) was loaded into human bone marrow MSCs (hBMSCs) derived exosomes to form Exo-PTEN (Guo et al., 2019). (Figures 4C, D) Researchers used Exo-PTEN by intranasal administration for the treatment of rats with transection of spinal cord T10 levels. Exo-PTEN could significantly accumulate in SCI lesion areas and co-localize in neurons at 24 h of administration, whereas, after administration to healthy rats, Exo-PTEN was predominantly distributed in the brain and olfactory bulb. They found that this targeting was derived from chemokine receptors on exosomes. The hypothesis of neuroinflammation-mediated chemotaxis was validated again. Surface-engineered EVs were also used for intranasal targeted drug delivery. Through lentiviral transfection, researchers obtained exosomes secreted by neuronal cells overexpressing Platelet Derived Growth Factor Alpha (PDGFA) on the membrane surface (Xiao et al., 2022). This exosome targets platelet-derived growth factor receptor α (PDGFRα)-positive oligodendrocyte progenitor cells (OPCs) and allows targeted delivery of montelukast to focal areas of myelin damage.

Obtaining sufficient EVs for clinical applications requires large numbers of MSCs and long-term culture. Recent studies have developed exosome-mimetic nanovesicles (NVs) to overcome these obstacles. NVs have particle size and compositions similar to exosomes and can transfer biomolecules from parental cells to recipient cells (Oh et al., 2015; Jo et al., 2016). NVs are produced by successive extrusion of cells cell membrane through micropore and nanopore filters. Hundreds of times more NVs can be obtained from the same number of cells than exosomes, and NVs contain a greater amount of both RNA and protein than the same number of exosomes (Jo et al., 2014). After treating hMSCs with PEGylated superparamagnetic iron oxide nanoparticles (IONPs) for 48 h, hMSC-derived exosome-mimetic nanovesicles (NV-IONP) can be obtained by centrifugation, extrusion, and magnetic separation (Kim et al., 2018). These NV-IONPs could converge more to the site of SCI in mice after tail vein injection by neodymium magnetic guidance, enhancing angiogenesis, reducing inflammation and apoptosis, and thus improving motor function. In addition to aiding NV-IONPs for targeted therapy, IONPs can also activate the JNK and c-Jun signaling cascades in hMSCs to boost therapeutic growth factors. To improve the enrichment and stagnation time of MSC-Exo at the injury site and enhance the targeting effect on the injury site, Researchers prepared umbilical cord blood-derived MSCs fused with macrophage membranes (MF-MSCs). Exosome-mimicking nanovesicles (MF-NVs) were obtained by extrusion and centrifugation (Lee et al., 2020). After tail vein injection, MF-NVs could be more targeted and accumulated in the injured spinal cord of mice and significantly improved motor function. This is attributed to the presence of functional proteins such as integrin-α4, integrin-β1, CD11b, and CD18 on the surface of macrophage membranes, which have an inflammatory propensity to increase the efficiency of MF-NVs in targeting the damaged spinal cord (Alon and Feigelson, 2002; Thamphiwatana et al., 2017). In conclusion, stem cell-derived exosomes demonstrated the most clinically applicable therapeutic effects in animal experiments. However, as with other lipid nanovesicles, the specific targeting and therapeutic mechanisms need to be further explored.

### 5.2 Macrophage-derived EVs

The microenvironment in the area of SCI has high levels of inflammatory factors that induce the recruitment of peripheral macrophages to injury sites. Activated macrophages are mainly the classically activated M1 phenotype with pro-inflammatory and neurocytotoxic effects, whereas the M2 phenotype is anti-inflammatory and neuroprotective (Zhou et al., 2020). M2-type macrophage-derived EVs inherit the inflammatory tropism and anti-inflammatory capacity of macrophages (Hamidzadeh et al., 2017). With this property, Gao et al. obtained Exos-Ber by incubating exosomes secreted by M2 macrophages with the anti-inflammatory drug Ber(Gao et al., 2021). They found that Exos-Ber was inflammatory-targeting to macrophages/microglia at the injury lesion after tail vein injection, and releasing Ber stably for 48 h. The natural drug, cur, was also loaded into macrophage exosomes (Ex-cur) by co-incubation for the treatment of cerebral ischemia (Lin et al., 2011; He et al., 2020). Ex-cur also has the advantage of inflammation-driven penetration of the BBB, as well as the ability to target NeuN+ cells and CD34^+^ cells in ischemic areas of the brain. Cur has also been used to co-load into Raw264.7-derived exosomes with superparamagnetic iron oxide nanoparticles (SPIONs). The exosome membranes were then conjugated with neurofelt protein-1 targeting peptide (RGERPPR, RGE) by click chemistry to obtain RGE-Exo-SPION/Cur (Jia et al., 2018). RGE is a specific ligand for NPR-1 and is highly targeted to glioma cells and tumor vascular endothelium, which will help exosomes become a powerful tool for targeted imaging and treatment of gliomas.

The yield of exosomes from M2 type macrophages is also limited, and the process of inducing macrophage conversion to M2 type requires expensive cytokines such as IL-4. The cell membranes of macrophages were similarly used to prepare cell-derived lipid nanovesicles. Continuous extrusion of macrophage membranes through polycarbonate membranes can obtain nanovesicles encapsulating NGFs (NGF-NVs). (Xia et al., 2021). Such NVs inherit the properties of macrophages, and the encapsulated NGF escapes clearance by the reticuloendothelial system (RES) and is thus more frequently delivered to SCI sites. The same method was also used to prepare macrophage-derived nanovesicles loaded with sodium alginate and naloxone (NAL-SA-MVs) using the same method (Liu et al., 2022). NAL-SA-MVs injected via tail vein will accumulate in large quantities in the SCI segments of mice and increase the concentration of SA and NAL, significantly promoting the recovery of motor function in SCI mice. The next step in the research will be the surface engineering of MVs to make the targeting ability more effective.

### 5.3 Microglia-derived EVs

Together with blood-derived monocyte-derived macrophages, microglia constitute the innate immune system of the central nervous system (Beck et al., 2010). Activated, proliferating microglia play a key role in scar formation following SCI. This multicellular interaction helps to isolate blood-derived immune cells at the core of the lesion, thereby protecting against non-inflammatory-mediated tissue injury leading to neuronal and oligodendrocyte damage (Bellver-Landete et al., 2019). Relatively little attention has been paid to microglia-derived EVs targeted for SCI therapy. Rat primary microglia treated with resveratrol can secrete resveratrol-loaded exosomes (Exo + RES), which can well resolve the solubility and bioavailability of resveratrol (Fan. et al., 2020b). Exo + RES shares with macrophage exosomes the same lymphocyte function-associated antigen-1 that can mediate lateral migration and help exosomes cross the BSCB. With the assistance of lymphocyte function-associated antigen-1, which mediates lateral migration and helps exosomes cross the BSCB, Exo + RES can be targeted to SCI segments in mice by intraperitoneal injection to obtain better motor function improvement. By lentiviral transfection of BV2 cells, EVs expressing milk fat globulin protein e8 (Mfg-e8) on the surface and loaded with IL-4 were isolated. With the “eat-me” signal of Mfg-e8, these EVs could target phagocytes in the area of neuroinflammatory disease lesions (Casella et al., 2018). Modification of the extracellular vesicles secreted by M2 microglia with the help of copper-free click chemistry can obtain Dual-EV, which is decorated with a vascular targeting peptide (DA7R peptide) and a stem cell recruitment factor (SDF) (Ruan et al., 2022). It has the ability to nest vascular endothelial cells and recruit NSCs, allowing for functional repair and neuronal regeneration of targeted damaged nerves. In the future, it will be a fine line of research direction regarding the comparison of EVs secreted by microglia from central origin or macrophages from peripheral origin in the targeted treatment of SCI.

### 5.4 Plasma EVs

Obtaining EVs without cell culture is another alternative way to increase yield, and large amounts of EVs with low immunogenicity can be obtained from autologous plasma (Kur et al., 2020). Plasma-derived EVs have been shown to cross the BBB and interact with TLR4 via surface-rich HSP70 to achieve ischemic brain targeting, effectively reducing ROS production and inhibiting mitochondrial apoptosis and BBB damage (Jiang et al., 2020b). To improve the delivery efficiency of growth-promoting peptides ILP and ISP to SCI, researchers loaded ILP, ISP, and RVG peptides onto the surface of autologous plasma exosomes (AP-EXOR&L&S) with the help of CP05 (Ran et al., 2022). RVG is a neuron-specific rabies virus glycoprotein with the ability to bind to the p75 neurotrophin receptor (p75NTR) and accelerate its retrograde axonal transport. With the help of RVG, AP-EXOR&L&S can target and migrate to the site of SCI and remain for more than 7 days for better neural repair. Another protein, GAP43, which increases in expression when neurons are damaged or stimulated (Sandelius et al., 2018), has also been used as a site for targeted therapy. Plasma-derived exos encapsulated with the drug Que, coupled with a monoclonal antibody to GAP43 (mAb GAP43) yields Que/mAb GAP43-Exo (Guo et al., 2021). After intravenous injection, Que/mAb GAP43-Exo can be targeted to accumulate at the brain injury lesion and slowly release Que. Since damaged neurons after SCI also express GAP43 in large amounts (Curtis et al., 1993), Que/mAb GAP43-Exo is also promising for targeted delivery of SCI.

### 5.5 Other cell-derived EVs

A series of other recent studies have also confirmed the therapeutic effects of other cell-derived EVs on SCI. For example, neuron-derived EVs promote spinal cord functional recovery by inhibiting the activation of M1 microglia and A1 astrocytes (Jiang et al., 2020a). GJA1-20 k-bearing exosomes secreted by astrocytes protect and restore mitochondrial function and downregulate the rate of apoptosis in damaged neurons (Chen et al., 2020). Pericyte-derived exosomes can protect the barrier of spinal microvascular endothelial cells under hypoxic conditions via the PTEN/AKT pathway (Yuan et al., 2019). Studies like these are numerous, but in-depth studies on the *in vivo* distribution of these EVs after administration and their ability to target SCI are needed. Researchers have tried more types of EVs for targeting brain diseases. Dendritic cells were transfected with plasmids encoding Lamp2b constructs, which in turn yielded dendritic cell-derived exosomes that specifically expressed Lamp2b on the membrane. GAPDH siRNA was then loaded into the exosomes using electroporation to obtain siRNA-RVG-EXO (Alvarez-Erviti et al., 2011). With the specific fusion of Lamp2b RVG peptide, siRNA-RVG-EXO can be targeted to aggregate in the brain. Therefore, there are many more types of cell-derived lipid nanovesicles that are worthy of engineering for targeted therapies of SCI as well as other diseases.

## 6 The prospect and future of lipid nanovesicles

In the field of targeted therapeutics, lipid nanovesicles have been extensively developed by researchers and they provide a biocompatible and engineerable tool for drug delivery. Synthetic lipid nanovesicles can be derived from natural or synthetic lipids, polysaccharides, sterols, or surfactants (Large et al., 2021). This classical lipid nanovesicles are relatively inexpensive to prepare and the process is well developed, but their simple surface structure makes them more likely to require additional modifications for targeted delivery capability and longer cycle times. On the other hand, instability of long-term storage and modification of multiple components to increase targeting efficiency poses a challenge for clinical translation (Tinkle et al., 2014; Hua et al., 2018). Intellectual property issues related to liposomes are also a perplexing factor at present. Therefore, more stable liposome storage devices and efficient surface modification methods need to be developed in the future, and clearer standards for patent protection of liposomes are needed. As latecomers, cell-derived lipid nanovesicles, especially EVs, are beginning to receive more attention from researchers. Their excellent biocompatibility and targeting capabilities make them promising for clinical applications. There are various ways to obtain them, including ultracentrifugation, polymer precipitation, immunoaffinity capture, size-exclusion chromatography, and microfluidic techniques (Dong et al., 2020). However, there is no recognized best method for achieving ‘high recovery, high specificity’ (Buschmann et al., 2021). Apart from this, their complex nature, low yields and batch-to-batch variability leave cell-derived lipid nanovesicles a long way from clinical application. Therefore, standardized preparation process to be established. Although different types of drugs can be co-incubated, electroporated, sonicated, repeatedly frozen and thawed, and genetically engineered to achieve drug loading, selecting suitable drugs and determining effective and safe doses is still a key issue. Researchers also need to further investigate the mechanisms by which lipid nanovesicles internalize drugs. Depending on how the different methods work, a convenient and stable loading method can be chosen for the therapeutic drug. Surface engineering modifications have recently been widely used to enhance the targeting of lipid nanovesicles. However, the structural and functional stability of the engineered modifications as well as the possibility of off-targeting need to be investigated in depth, and more effective receptor and ligand combinations need to be developed. Here, comparing the advantages and disadvantages of cell-derived versus synthetic lipid nanovesicles is a contradictory topic, as they are both members of nanovesicles. For example, the lipid component of liposomes can be used in exosomes to achieve higher intelligence regarding membrane fusion (Sato et al., 2016; Fernandes et al., 2021). Liposomes can also be modified with the help of cell membranes to obtain properties that mimic EVs(Tang et al., 2021). Therefore, future researchers need continue to explore new strategies to prepare lipid nanovesicles with higher yields, purity, stability, and biocompatibility. More importantly, issues common to lipid nanovesicles, such as dosage and frequency of administration, off-target potential, unstable production and preservation, and still many targeting and therapeutic mechanisms that need to be clarified, should be the focus of future research.

Despite the challenges, lipid nanovesicles are already in clinical trials and have shown good biocompatibility and therapeutic potential thanks to the efforts of researchers (Surana et al., 2022). Breakthroughs are also beginning to occur in the treatment of diseases of the central nervous system, especially the brain (Wang et al., 2020; Bashyal et al., 2022). Although research into targeted treatments for SCI has been relatively slow to progress, many research teams and pharmaceutical companies have been actively promoting the clinical use of relevant products. Many recent studies on lipid nanovesicles for the targeted treatment of SCI have involved drug loading and surface modifications. Engineered EVs targeting SCI have also entered preclinical studies (NurExone, 2022). It is reasonable to expect that a safe, stable, and effective targeted treatment system for spinal cord injury will be developed in the future.

## 7 Conclusion

The use of lipid nanovesicles for targeted therapies has great potential and they have been investigated in drug-delivery therapies for a variety of diseases. Plenty of lipid nanovesicles research offers promising therapeutic approaches for the targeted treatment of SCI. Either the lipid nanovesicles themselves or the engineering modifications to them have a broad space for exploitation in the future. However, despite the encouraging repair results achieved in most recent studies, many issues remain to be addressed, especially in terms of clinical translation. Finally, it is also important to emphasize that due to the complex pathophysiological mechanisms of SCI, it is unlikely that a single treatment approach can overcome multiple barriers. In addition to targeted therapy, other treatment strategies such as traditional surgery and medication, electro-neuromodulation, rehabilitation, and bio-tissue engineering should be combined. A multidisciplinary approach is needed if we want to win the battle against paralysis.

## Figures and Tables

**Table:** 

**SCI**	Spinal cord injury
**EVs**	Extracellular vesicles
**BSCB**	blood-spinal cord barrier
**BBB**	blood-brain barrier
**CL**	positively charged liposomes
**AL**	negatively charged liposomes
**pOXR1**	Oxidation resistance 1 plasmid
**RGD**	arginine-glycine-aspartate
**CAQK**	cysteine-alanine-glutamine-lysine
**EDC**	1-(3-Dimethylaminopropyl)-3-ethylcarbodiimide hydrochloride
**NHS**	N-Hydroxysuccinimide
**ILP**	insulin like peptide
**ISP**	insulin-stimulating peptide
**RVG**	rabies virus glycoprotein
**CP05**	Cys-Arg-His-Ser-Gln-Met-Thr-Val-Thr-Ser-Arg-Leu
**TAT**	trans-activated transducing
**PEG**	polyethylene glycol
**PLGA**	Poly (lactid-glycolide acid)
**PEG-OQCMC**	PEGylated amphiphilic octadecyl quaternised carboxymethyl chitosan
**Chol**	Cholesterol
**SD**	Sprague-Dawley
**CsA**	Cyclosporine A
**NPs**	nanoparticles
**OQLCS =**	octadecyl-quaternized lysine modified chitosan
**PC, Phosphatidylcholine; mPEG 2000-DSPE**	N-(Carbonyl-methoxypolyethylene glycol 2000)-1,2-distearoyl-sn-glycerol-3-phosphoethanolamine
**MH**	minocycline hydrochloride
**DS**	dextran sulfate
**BDNF**	brain-derived neurotrophic factor
**DOTAP**	(2,3-Dioleoyloxy-propyl)-trimethylammonium-chloride
**DOPC**	1,2-dioleoyl-sn-glycero-3-phosphocholine
**DOPS**	1, 2-dioleoyl-sn-glycero-3-phospho-L-serine
**DSPC**	1,2-Dioctadecanoyl-sn-glycero-3-phophocholine
**CTB**	cholera toxin B
**R8**	Octa-arginine
**HSPC**	1,2-Diacyl-sn-Glycero-3-Phosphocholine
**COL**	chitosan oligosaccharide lactate
**Se-apamin**	diseleno bond-stabilized apamin
**CUR**	curcumin
**BMSCs**	bone marrow stromal cells
**miR**	microRNA
**HucMSCs**	human umbilical cord mesenchymal stem cells
**ADSCs**	adipose stem cells
**CSPG**	Chondroitin sulfate proteoglycan
**iNSCs**	induced neural stem cells
**siRNA**	small interfering RNA
**hBMSCs**	human bone marrow stem cells
**PTEN**	phosphatase and tensin homolog
**IONPs**	iron oxide nanoparticles
**MF-NVs**	macrophage-derived nan-ovesicles
**Ber**	berberine
**NGF**	nerve growth factor
**SA**	sodium alginate
**NAL**	naloxone
**Res**	resveratrol
**p75NTR**	neurotrophin receptor p75
